# Somatic embryogenesis of Arabica coffee in temporary immersion culture: Advances, limitations, and perspectives for mass propagation of selected genotypes

**DOI:** 10.3389/fpls.2022.994578

**Published:** 2022-10-06

**Authors:** María Elena Aguilar, Xiao-yang Wang, Maritza Escalona, Lin Yan, Li-fang Huang

**Affiliations:** ^1^ Biotechnology Laboratories, Tropical Agricultural Research and Higher Education Center (CATIE), Turrialba, Costa Rica; ^2^ Spice and Beverage Research Institute, Chinese Academy of Tropical Agricultural Sciences (CATAS), Wanning, China; ^3^ Key Laboratory of Genetic Resources Utilization of Spice and Beverage Crops, Ministry of Agriculture and Rural Affairs, Wanning, China; ^4^ Hainan Provincial Key Laboratory of Genetic Improvement and Quality Regulation for Tropical Spice and Beverage Crops, Wanning, China; ^5^ Plant Tissues Culture Lab, Centro de Bioplantas, Universidad Ciego de Ávila, Ciego de Ávila, Cuba

**Keywords:** somatic embryogenesis, temporary immersion culture, semi-automation micropropagation, coffee, *Coffea arabica*

## Abstract

Culture in temporary immersion systems (TIS) is a valuable tool for the semi-automation of high frequency somatic embryogenesis of coffee. This system allows the intermittent exposure of explants to liquid medium in cycles of specific frequency and duration of immersion with renewal of the culture atmosphere in each cycle. TIS have revolutionized somatic embryogenesis of coffee plants as an alternative for scaling up and reducing costs associated with labor-intensive solid media culture. In Central America, somatic embryogenesis is employed on a commercial scale to produce F1 *Coffea arabica* hybrids. In Asia and Africa, somatic embryogenesis is used for the multiplication of selected genotypes of *C. arabica* and *C.canephora*. Somatic embryogenesis of coffee plants is considered a model system for woody species due to its biological versatility and low frequency of somaclonal variation. Nevertheless, the success of somatic embryogenesis for mass propagation of coffee plants depends on the development, optimization, and transfer of complementary technologies. Temporary immersion using the RITA^®^ bioreactor is, so far, the best complementary tool for somatic embryogenesis of Arabica coffee for a single recipient with simple changes in liquid media. Likewise, high volume bioreactors, such as 10-L glass BIT^®^ and 10-L flexible disposable plastic bags, have been successfully used for somatic embryogenesis of other coffee species. These bioreactors allow the manipulation of thousands of embryos under semi-automated conditions. The protocols, advantages, and benefits of this technology have been well documented for organogenesis and somatic embryogenesis pathways. However, adaptation in commercial laboratories requires technical and logistical adjustments based on the biological response of the cultures as well as the costs of implementation and production. This review presents the historical and present background of TIS and its commercial application and, in particular, pertinent information regarding temporary immersion culture for *C. arabica* somatic embryogenesis. The main limitations of this technology, such as hyperhydricity, asynchrony, and developmental abnormalities, are examined, and a critical analysis of current knowledge regarding physiological, biochemical, and molecular aspects of the plant response to temporary immersion is offered. Further, perspectives are provided for understanding and solving the morpho-physiological problems associated with temporary immersion culture of coffee plants.

Systematic Review Registration:

## 1 Introduction

*Coffea arabica* was introduced to Latin America in the 18^th^ century, and the region accounts for 80% of the world’s production of this coffee. *C. arabica* is one of the main sources of income for many countries (Bertrand et al., 2011). According to the International Coffee Organization (https://www.ico.org/Market-Report-21-22-e.asp), the global coffee production has increased by more than 60% since 1990. The Arabica-to-Robusta ratio is approximately 60/40, and only 30% of the produce is consumed in coffee-producing countries. Therefore, coffee remains a basic export product.

*C. arabica* varieties grown in Latin America have a very narrow genetic base because they are derived from genealogical selections from a few individuals (Bertrand et al., 2005). Given the economic importance of Arabica coffee for Central America and the presence of the international coffee germplasm collection at the Tropical Agricultural Research and Higher Education Center (*Centro Agronómico Tropical de Investigación y Enseñanza* – CATIE), in Costa Rica, a Regional Genetic Improvement Program was initiated, involving the French Agricultural Research Center for International Development (*Centre de Coopération Internationale en Recherche Agronomique pour le Développement* – CIRAD), and regional coffee institutes. The program aimed at increasing the genetic variability of this species and developing more vigorous F1 hybrids than the currently grown varieties, with optimal agronomic characteristics such as disease resistance and cup quality (Bertrand et al., 2005). Albeit autogamous, *C. arabica* is highly heterozygous (Srinivasan and Vishveshvara, 1978); consequently, these hybrids can only be propagated vegetatively; therefore, since their creation, they have been propagated *via* somatic embryogenesis (SE) (Etienne and Bertrand, 2001).

SE is a high-efficiency propagation technique for early genetic gains through rapid and large-scale diffusion of elite individuals (Etienne et al., 2012). Crop production by SE is an intensive, slow, and expensive process, in contrast to plants grown from seed (Aguilar et al., 2017). Technical innovations such as embryogenic cell suspensions (ECS), SE in temporary immersion bioreactors (RITA^®^) (Berthouly et al., 1995; Etienne et al., 1997a), and direct somatic embryo sowing in the greenhouse have improved the coffee SE technique (Etienne-Barry et al., 1999; Barry-Etienne et al., 2002). Recently, complementing SE with classic vegetative propagation techniques such as root cuttings from somaclones has improved hybrid multiplication and lowered production costs (Aguilar et al., 2018; Etienne et al., 2018).

After half a century of research and innovation, coffee SE has become a model for the SE of woody species and one of the few successful examples of propagating elite genotypes on a commercial scale. Unresolved technical problems, coupled with high production costs and the lack of promotion and financing policies, have limited the distribution of these hybrids in Central America for more than a decade (Aguilar et al., 2017). However, millions of coffee plants have been produced by this technology, the majority planted on commercial farms in Latin America, Africa and Asia (Etienne et al., 2018).

Nevertheless, new scientific approaches should be developed to increase the efficiency of *C. arabica* SE. Knowledge derived from tools such as epigenetics and omics will help decipher the complex mechanisms that regulate SE and optimize protocols with an increased scientific focus and impact.

This review aims at summarizing knowledge on *C. arabica* SE since its inception and analyzing the main innovations and their impact on the multiplication of elite plants. Given the importance of temporary immersion cultivation for coffee SE and micropropagation on a commercial scale, the evolution and application of this technology is reviewed, showing the main cultivation limitations of coffee SE in RITA^®^ bioreactors. Due to the lack of scientific information on physiological changes related to *C. arabica* SE in RITA^®^ bioreactors and the absence of an ecophysiological characterization of the culture environment in the bioreactor, studies on other species are included in this review to compare and understand explant responses to temporary immersion cultivation. The causes, manifestations, and possible solutions to different physiological and morphological disorders, such as hyperhydricity and asynchronous development, or genetic and epigenetic disorders, such as somaclonal variation (SV), are also presented in this review.

In addition, this review outlines how simple strategies have solved most problems associated with low germination and plant conversion rates under *in vitro* conditions while increasing hybrid multiplication and their scaling in the greenhouse at a reduced cost. However, these materials can only be transferred to the smallholder farmer if coffee-governing institutions commit to finding the necessary technical and financial mechanisms for facilitating this process.

## 2 SE: A powerful tool for clonal propagation of elite materials

### 2.1 General aspects of SE

SE is a process in which somatic cells can dedifferentiate into totipotent cells under appropriate conditions and reprogram their development towards the embryogenic pathway with the appropriate stimulus (Fehér et al., 2003; Jiménez, 2005; Karami et al., 2009; Guan et al., 2016; Aguilar-Hernández and Loyola-Vargas, 2018). A somatic embryo is a bipolar structure resembling a zygotic embryo, which develops from a somatic cell without vascular connection to the original tissue (Von Arnold et al., 2002). This bipolarity differentiates somatic embryos from *in vitro*-regenerated adventitious organs (shoots or roots), which are unipolar and have a vascular connection to the tissue of origin (Horstman et al., 2017).

SE is a unique experimental model for understanding the molecular and cellular bases of the development plasticity of a plant (Fehér et al., 2003). However, SE is a complex paradigm in the biology of plant development given the complexity of its regulatory mechanisms (Smertenko and Bozhkov, 2014). Comparative studies between zygotic embryogenesis (ZE) and SE of model species have deciphered numerous regulatory mechanisms and established similarities and differences between both embryogenesis processes. These processes share different morphogenetic states during embryo development in dicots, monocots, and gymnosperms (Zimmerman, 1993; Dodeman et al., 1997; Von Arnold et al., 2002; Fehér et al., 2003). Common regulatory mechanisms seem to be involved in the early globular embryo stages, in addition to signaling molecules, such as plant growth regulators (PGRs), which play a key role in the development of both types of embryos (Dodeman et al., 1997). ZE starts from a single cell and reaches the globular state consisting of a specific number of cells; SE starts from a cell or a group of cells and forms a globular structure with variable number of cells (Smertenko and Bozhkov, 2014). During the first zygote divisions or during early SE, the suspensor is formed in the basal embryo, a multicellular organ that determines the apical-basal polarity of the embryo (Zimmerman, 1993). Once differentiated, this organ is eliminated through the vacuole by programmed cell death (PCD), promoting cell proliferation at the apical end until the seedling is formed (Smertenko and Bozhkov, 2014). The absence of the endosperm in the somatic embryo, integuments, and desiccation and dormancy periods mark key differences in the processes, given their importance in zygotic embryo conservation and maturation during germination (Dodeman et al., 1997; Fehér et al., 2003).

SE encompasses two processes—induction and expression—which are independent of each other and influenced by different factors (Jiménez, 2005). Induction is the fundamental difference between ZE and SE because somatic cells need physical and chemical stimuli to acquire embryogenic competence (Dodeman et al., 1997; Fehér et al., 2003). Exogenous auxins are decisive during induction (Nomura and Komamine, 1985), given their role in cell dedifferentiation and in the transition of a somatic cell to the embryogenic state (Dodeman et al., 1997). During this period, the cells are exposed to variable physiological and stress conditions (exogenous PGRs or mechanical stress), dedifferentiating, reprogramming their gene expression, and inducing changes in their morphology, physiology and metabolism depending on their adaptability (Fehér et al., 2003; Namasivayam, 2007; Karami et al., 2009). The frequency of induction depends on the genotype, explant development stage (Namasivayam, 2007), and endogenous hormone levels (Jiménez, 2005).

During expression, embryogenic cells do not require external stimuli (auxins) to differentiate into somatic embryos (Nomura and Komamine, 1985; Yang and Zhang, 2010); however, stress conditions may trigger the process (Fehér et al., 2003). Embryogenic cells commonly appear as proembryogenic masses (PEMs) composed of small, isodiametric cells with a dense cytoplasm (Dodeman et al., 1997; Menéndez-Yuffá and García de García, 1997), a prominent nucleus and nucleolus, small vacuoles, and abundant starch granules, suggesting that they are cells with intense RNA synthesis and metabolic activity (Williams and Maheswaran, 1986).

In contrast to direct or low-frequency SE (LFSE), which originates directly from the explant without intermediate callus formation, indirect or high-frequency SE (HFSE) is mediated by callus formation (Williams and Maheswaran, 1986). HFSE is a multi-step regeneration process, which begins with the formation of PEMs and continues with the regeneration, maturation, and conversion of embryos into plants (Von Arnold et al., 2002). These embryogenesis mechanisms can occur simultaneously in the same explant, thereby hindering their differentiation (Guan et al., 2016).

SE is a tool with enormous potential for elite genotype multiplication on a commercial scale given the cell regeneration capacity and high proliferation rates of such genotypes, combined with the possibility of growing them in liquid medium, automating these cultures in bioreactors and planting them as synthetic seeds (Guan et al., 2016; Horstman et al., 2017). In genetic improvement, SE enables the multiplication of elite materials for evaluation purposes, shortening improvement cycles (Horstman et al., 2017; Pais, 2019), as well as plant regeneration during genetic transformation and somatic hybridization (Karami et al., 2009; Guan et al., 2016; Horstman et al., 2017), and cryopreservation and storage of embryogenic cultures for long-term clone evaluation (Aguilar et al., 1993; Engelmann et al., 1994; Florin et al., 1995; Egertsdotter et al., 2019). Hence, SE has been used for numerous agricultural species such as coffee (Zamarripa et al., 1991; Van Boxtel and Berthouly, 1996; Etienne et al., 1997a), cocoa (Aguilar et al., 1992; López-Báez et al., 1993; Alemanno et al., 1996; Maximova et al., 2002; Niemenak et al., 2008), and citrus (Tomaz et al., 2001; Pan et al., 2009), and forest species, including conifers (Lelu-Walter et al., 2006; Montalbán et al., 2012; Kim, 2015; Maruyama and Hosoi, 2019) and tropical and temperate angiosperms (Pintos et al., 2008; Lardet et al., 2009; Correia et al., 2012; Corredoira et al., 2015; Mignon and Werbrouck, 2018). However, only a few species have reproducible protocols for large-scale propagation and commercial planting (Etienne and Bertrand, 2001; Ducos et al., 2007a; Ducos et al., 2007c; Maximova et al., 2008; Etienne et al., 2012; Etienne et al., 2018). For most species, the commercial application of SE has many limitations associated with the genotype (Pais, 2019), low quality of the embryos, problems with embryo maturation and conversion into a plant (Jiménez, 2005), somaclonal variation (SV), and lack of reproducibility of the process (Etienne et al., 2012).

### 2.2 SE of coffee: A historical background

Since SE was first reported for Robusta coffee (Staritsky, 1970), different coffee species and their varieties (Söndahl and Sharp, 1977; Zamarripa et al., 1991; Van Boxtel and Berthouly, 1996; De Feria et al., 2003; Giridhar et al., 2004), clones (Ducos et al., 2003; Santana et al., 2004), and hybrids (Etienne et al., 1997a; Etienne and Bertrand, 2001; Etienne and Bertrand, 2003; Etienne et al., 2012; Aguilar et al., 2018) have been evaluated for their embryogenic competence. Stem (Staritsky, 1970; Nassuth et al., 1980) and leaf (Söndahl and Sharp, 1977; Dublin, 1981; Yasuda et al., 1985; Berthouly and Michaux-Ferriere, 1996; Hatanaka et al., 1999) explants and parts of zygotic embryos (Sreenath et al., 1995) were evaluated. Direct and indirect SE were first observed in *C. arabica* var. *Bourbon* leaves by Söndahl and Sharp (1977).

Subsequently, the culture conditions were evaluated (Söndahl and Sharp, 1977; Samson et al., 2006), in addition to assessing the effects of PGRs (Van Boxtel and Berthouly, 1996; Samson et al., 2006) and genotypes (Van Boxtel and Berthouly, 1996; Etienne et al., 1997a; Molina et al., 2002; Etienne et al., 2012). Histological analyses were also performed to determine the origin and development of somatic embryos (Michaux-Ferrière et al., 1987; Michaux-Ferrière et al., 1989; Menéndez-Yuffá and García de García, 1997).

Liquid medium cultures (Zamarripa et al., 1991; Van Boxtel and Berthouly, 1996), embryogenic cell suspensions (ECSs) (Zamarripa et al., 1991; Van Boxtel and Berthouly, 1996), and continuous (Zamarripa et al., 1991; De Feria et al., 2003; Ducos et al., 2007b) and temporary (Etienne et al., 1997a; Albarrán et al., 2005; Ducos et al., 2007a; Ducos et al., 2007b) immersion bioreactors considerably advanced propagation to a commercial scale. According to Etienne et al. (2018) this technology has allowed the industrialization and commercialization of SE coffee for more than 15 years. More than 20 million plants of *C. arabica* have been distributed in Central America in the last 10 years. In addition, Nestlé projected a worldwide distribution of 220 million plants of both crops by 2020. Apart from hundreds of plants that have been distributed by smaller laboratories.

Innovations such as directly sowing embryos in a greenhouse (Etienne-Barry et al., 1999; Barry-Etienne et al., 2002), rooting cuttings from somatic embryos (Mesén and Jiménez, 2016; Georget et al., 2017), and studying SV (Etienne and Bertrand, 2001; Etienne and Bertrand, 2003; Menéndez-Yuffá et al., 2010; Bobadilla Landey et al., 2013; Bobadilla Landey et al., 2015; Muniswamy et al., 2017) and the agronomic performance of SE plants (Ducos et al., 2003; Bertrand et al., 2005; Bertrand et al., 2011; Marie et al., 2020) have led to the successful scaling of elite coffee genotypes.

Simultaneously, techniques such as cryopreservation (Florin et al., 1995; Bertrand-Desbrunais et al., 1998), genetic transformation (Van Boxtel et al., 1995; Hatanaka et al., 1999; Quiroz-Figueroa et al., 2002; Rosillo et al., 2003; Gatica-Arias et al., 2008; Ribas et al., 2011), and protoplast culture (Schöpke et al., 1987; Acuña and De Peña, 1991; Toruan-Mathius, 1992; Tahara et al., 1994) have harnessed the benefits of HFSE and ECS culture for their regeneration processes.

Technologies such as epigenetics (Bobadilla Landey et al., 2013; Bobadilla Landey et al., 2015; Etienne et al., 2016) and omics (Arroyo-Herrera et al., 2008; Mukul-López et al., 2012; Nic-Can et al., 2013; Silva et al., 2013; Silva et al., 2014; Freitas et al., 2017; Awada et al., 2019) are revolutionizing knowledge on the cellular, molecular, genetic, and epigenetic mechanisms that regulate coffee SE. Genomics (Karami et al., 2009), and proteomics (Guan et al., 2016) have made it possible to identify a large number of genes and proteins involved in different cellular processes of SE. The Brazilian Coffee Genome Project (Vieira et al., 2006) achieved the sequencing of 214.964 ESTs (Expressed Sequence Tags) in genes from different tissues including embryogenic calli and zygotic embryos of *C. arabica*, *C. canephora* and *C. racemosa*. Sequences potentially involved in SE processes were identified and could be used to develop molecular markers in order to increase the methodological efficiency of coffee SE protocols (Silva et al., 2013; Silva et al., 2015). Homologous sequences of genes linked to different phases of SE in other species [LEAFY COTYLEDON (LEC1/LEC2), WUSCHEL-RELATED HOMEOBOX (WUS), SE RECEPTOR KINASE (SERK), BABY BOOM (BBM)] have been identified in coffee (Campos et al., 2017). LEC expression in coffee was observed after SE induction, therefore, it is considered essential during embryo maturation (Nic-Can et al., 2013). They showed that the embryogenic capacity of *C. canephora* regulated by the LEC1, BBM1 and WUS WOX4 genes is under epigenetic control. The initiation of SE, cell differentiation and embryogenic development are regulated by epigenetic mechanisms, such as DNA methylation and histone modification.

A WUS heterologous promoter of embryogenic induction in somatic cells and embryo differentiation was identified in *C. canephora* (Arroyo-Herrera et al., 2008; Silva et al., 2013). The homologous sequences CaSERK (Silva et al., 2014) and CaBBM (Silva et al., 2015) were found in EC and ECS of *C. arabica*. Similarly, Ethylene Response Factor (ERF) genes associated mainly with biotic and abiotic stresses have also been observed in EC and ECS of coffee and other species (Daude et al., 2020). Variations in the quality of the ECS over time were observed by the decrease in the expression of these genes and associated with the appearance of non-embryogenic regions after 60 days of culture (Torres et al., 2015). Finally, the validation of reference genes for SE of *C. arabica* has also been studied by means of Quantitative real-time PCR (qPCR) analysis (Freitas et al., 2017).

In addition, genome editing technologies based on highly efficient genetic transformation and plant regeneration systems offer great opportunities for functional genomics and molecular breeding (Etienne et al., 2018). CRISPR/Cas9 technology has been successfully used to introduce mutations into the coffee genome (Breitler et al., 2018).

Proteomic studies on SE have been performed in numerous species including coffee (Tonietto et al., 2012; Campos et al., 2016). Proteomic profiles allowed to identify specific proteins for different phases of coffee SE with potential as molecular markers. Proteins such as enolases, could be used as markers of the different phases of coffee SE (Tonietto et al., 2012); others such as 11S storage globulins may be useful for the identification of embryogenic and non-embryogenic genotypes (Tonietto et al., 2012). Mukul-López et al. (2012) evaluated the extracellular proteomic profile of cell suspensions of *C. arabica* and *C. canephora*, in cell proliferation culture and during SE induction. In both species, specific proteins of the embryogenic states and others typical of the non-embryogenic condition were observed.

In addition, the elaboration of metabolomic and hormonal profiles in each phase of the SE of coffee has allowed to characterize the cellular metabolism and its interaction with the hormonal mechanisms, as already mentioned (Awada et al., 2019).

Molecular markers of coffee are also used to analyze the genetic and epigenetic stability of DNA from plants regenerated *via* SE (Bobadilla Landey et al., 2013; Bobadilla Landey et al., 2015; Muniswamy et al., 2017).

### 2.3 Features of SE of *C. arabica*

#### 2.3.1 Development phases and influencing factors

*C. arabica* SE consists of different growth phases, defined by the composition and nature of the culture media (i.e., whether the medium is solid or liquid), with continuous agitation or temporary immersion, and by the presence or absence of light. These conditions have been improved to increase the embryogenic response of different genotypes (Van Boxtel and Berthouly, 1996; Etienne et al., 1997a; Etienne-Barry et al., 1999; Albarrán et al., 2005; Samson et al., 2006; Etienne et al., 2013).

**Figure 1:**
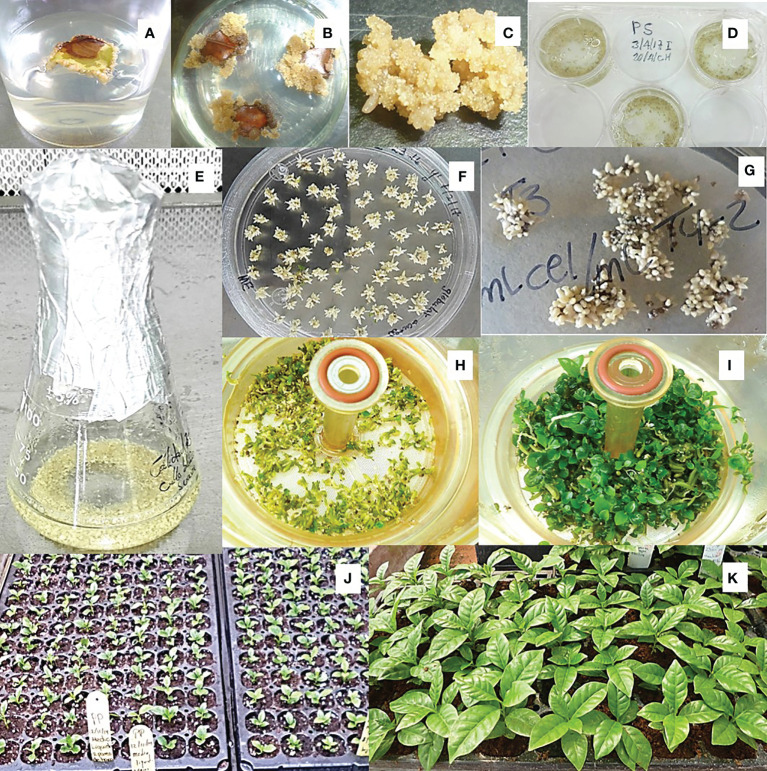
Induction and multiplication of EC and regeneration of somatic embryos. **(A)** Explant after one month of culture in darkness in the T1 medium, showing callus cicatrization; **(B)** brown color explants showing the EC; **(C)** mass of friable EC; **(D)** initiation of cellular suspensions in multi-cavity dishes with liquid T3 medium; **(E)** establishment of ECS in Erlenmeyer flasks; **(F)** regeneration of EC on semi-solid Yasuda medium; **(G)** regeneration of ECS on filter paper in semi-solid T4 medium; **(H)** torpedo embryos in the germination phase at RITA^®^; **(I)** embryos in conversion phase in RITA^®^; **(J)** plants starting the acclimatization phase; **(K)** plants at the end of the acclimatization phase.

**Table 1** presents *C. arabica* SE divided into five laboratory phases and one greenhouse phase (Aguilar et al., 2018). The culture media are outlined in **Table 2**. The Murashige and Skoog (1962) medium is used in most of the processes, except for embryo regeneration, for which the Yasuda et al. (1985) medium is used. The morphogenetic events that characterize each phase are shown in **Figure 1** (Aguilar et al., 2018).

**Table 1:** Phases of *Coffea arabica* somatic embryogenesis, culture conditions, duration and biological responses associated with each phase.

Phases	Media and culture vessel				Duration(Months)	Remarks
	Solid	Liquid	Temporary immersion	Photoperiod		
1. Callus induction	Vials 10 ml			Dark	1	Explants form scar calluses
2. Embryogenic calluses proliferation	vessels 20 ml			Indirect light	6-10	Explants turn black, visible embryogenic callus formation
3. Embryogenic callus Multiplication (ECS)		Erlenmeyer flasks		Light	6	Multiplication of cellular aggregates
4. Somatic embryos regeneration	Petri dishes	Erlenmeyer flasks	RITA^®^	Indirect light	2.5	White embryos in torpedo state
5. Somatic embryos germination and conversion	Vessels^1^ 250 ml		RITA®21L	Light	3-5	Embryos elongate, turn green, develop cotyledons and their first true leaves and roots
6. Acclimatization	Plastic trays			Light	4	Development of plantlets, numerous leaves and roots

^1^Somatic embryos germination and conversion on solid medium: 3 months.

^2^Somatic embryos germination and conversion into RITA^®^: 5 months.

**Table 2:** Culture media components used in each phase of *C. arabica* somatic embryogenesis, including mineral salts, vitamins, growth regulators, organic additives and pH.

Component	Culture media					
	T1	T2	T3	T4	T5	Yasuda
	**%**					
MS macroelements	50	50	50	50	100	25
MS microelements	50	50	50	50	100	50 ^1^
MS FE-EDTA	50	50	50	50	100	50
Morel vitamin					100	
	**mg/L**					
KH2PO4						42.5
Cysteine		40	10			
Thiamine	10	20	5	10		10
Glycine	1	20		2		
Nicotinic acid	1		0.5	1		1
Pyridoxine	1		0.5	1		1
Myo-inositol	100	200	100	200		100
Hydrolyzed casein	100	200	100	400		
Malt extract	400	800	200	400		
Adenine sulfate		60		40		
2,4-D (2,4-Dichlorophenoxyacetic acid)	0.5	1	1			
IBA (Indole-3-butyric acid)	1					
2 iP (N6-(2-Isopentenyl adenine)	2					
BAP (6-Benzylaminopurine)		4		2	0.3	1
Kinetin			1			
	**g/L**					
Sucrose	30	30	15	40	40	30
Phytagel ^2^	2.2	2.2		2.2	2.2	2.2
pH	5.6	5.6	5.6	5.6	5.6	5.6

^1^Yasuda microelements: 3.1 mg/L H_3_BO_3_, 11.2 mg/L MnSO_4_.7H_2_O, 4.3mg/L ZnSO_4_.7H_2_O, 0.125mg/L.

Na_2_MoO_4_.2H_2_O, 0.05 mg/L CuSO_4_.5H_2_O.

^2^Semi-solid culture media.

#### 2.3.2 Embryogenic callus induction

The explant, genotype, culture media composition, and *in vitro* culture environment are some of the factors that affect *C. arabica* SE (Campos et al., 2017). Immature leaves are the best explants for inducing SE in this species (Van Boxtel and Berthouly, 1996). Other factors associated with the embryogenic response depend on the growth conditions of the mother plant (Berthouly and Michaux-Ferriere, 1996), explant collection month (Molina et al., 2002), and physiological state of the donor plant (Campos et al., 2017).

Considering the variability in EC formation among genotypes, Arabica coffee SE is a genotype-dependent process. The genotype has a strong effect on the induction of both (low- and high-frequency) embryogenic pathways (Van Boxtel and Berthouly, 1996; Molina et al., 2002). This effect was also observed in *C. arabica* and *C. canephora* cultivars as a function of explant type (Giridhar et al., 2004) or culture medium composition (Rojas-Lorz et al., 2019). F1 hybrids are also genotype dependent, with differences in the embryogenic explant frequency and EC quality between genotypes (Etienne et al., 1997a). Depending on the hybrid, only 10–40% explants with a healing callus produce embryonic calluses (Etienne et al., 2012). This effect is carried over to the other development phases, and low-embryogenic hybrids are recalcitrant (Etienne et al., 1997a). The time of EC onset varies among F1 hybrids; some produce large quantities between 4 and 6 months, whereas others are more recalcitrant and take between 8 and 10 months to produce embryonic calluses (Aguilar et al., 2017). The frequency of SV is also genotype dependent in this species (Etienne and Bertrand, 2001; Etienne and Bertrand, 2003; Campos et al., 2017).

Söndahl and Sharp (1977) achieved HFSE in *C. arabica* through a two-step process: first, a culture was performed with auxins for explant conditioning and primary callus induction; and second, an auxin-free culture was performed for EC formation. HFSE is more widely used for its high yield in embryo production (Berthouly and Michaux-Ferriere, 1996) and for the possibility of using liquid medium for the mass production of embryos (Neuenschwander and Baumann, 1992; Van Boxtel and Berthouly, 1996). Changes in the concentration of culture medium components and PGRs (auxins and cytokinins) improved EC yield and production time (Van Boxtel and Berthouly, 1996; Samson et al., 2006).

#### 2.3.3 Importance of establishing embryogenic cell suspensions

Once the EC was obtained, its multiplication in ECS was the main goal (Van Boxtel and Berthouly, 1996; Etienne et al., 1997a; De Feria et al., 2003). Van Boxtel and Berthouly (1996) achieved high EC multiplication rates in a modified liquid medium with a high (4.5 µM) concentration of 2,4-D (2,4-dichlorophenoxyacetic acid) and subcultures with fresh medium every 7 or 10 days. However, the impact of high concentrations of 2,4-D on the SV forced them to reduce the concentration of auxins and the culture time (Etienne et al., 2012; Bobadilla Landey et al., 2013; Bobadilla Landey et al., 2015). Cell suspensions enable EC multiplication and maintenance, the development of regeneration cycles, and the inoculation of large volumes of EC during the regeneration and conversion phases in liquid medium. Approximately 1 L of undifferentiated tissue is sufficient to inoculate 20−30 L of embryogenic culture at a density of 1 g FW L^-1^, and each gram can produce up to 50 thousand plantlets (Ducos et al., 2007b). ECS are very useful for EC multiplication in poorly embryogenic genotypes (Aguilar et al., 2017; Aguilar et al., 2018). In addition, cell lines of interest can be stored and preserved in liquid nitrogen (Florin et al., 1995).

#### 2.3.4 Pathways for regeneration, germination, and conversion to plants

Van Boxtel and Berthouly (1996) used liquid medium followed by mature embryo transfer to solid germination medium for ECS regeneration, while Etienne et al. (1997a) used solid medium for ECS regeneration. ECS of F1 hybrids was regenerated on Yasuda or T4 solid media (Aguilar et al., 2018). In hybrids that produce a considerable number of embryonic calluses, regeneration on solid media is a quick method (2−2.5 months) for developing high-quality torpedo embryos. Addition of PGRs and other substances to solid or liquid media accelerates regeneration rates (Papanastasiou et al., 2008) and improves embryo quality (Quiroz-Figueroa et al., 2001).

**Figure 2:**
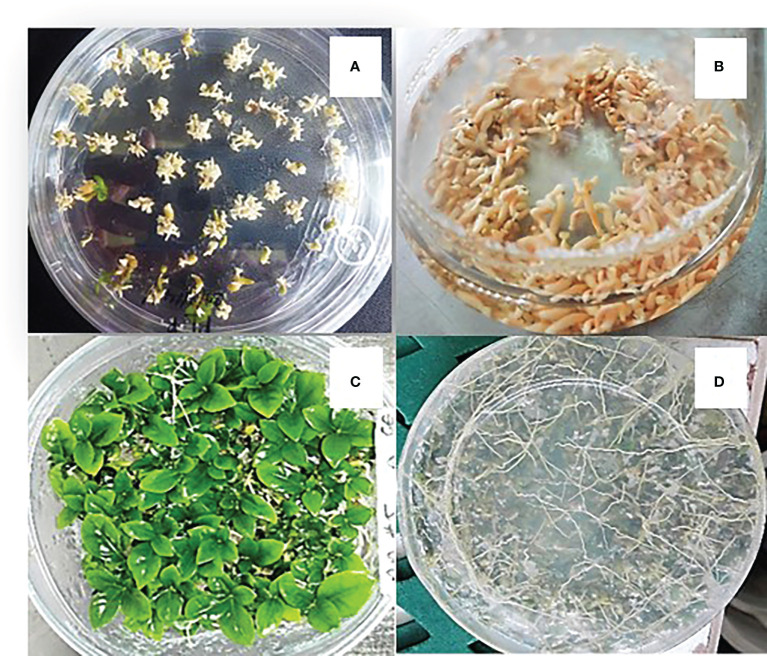
Germination of somatic embryos and conversion into plants in semi-solid T5 medium. **(A)** Regeneration of EC on semi-solid Yasuda medium; **(B)** pre-germination of embryos regenerated in liquid T5 medium in Erlenmeyer flasks; **(C)** conversion into plants in semi-solid T5 medium; **(D)** plants showing root development.

Embryo pre-germination in liquid medium was used as a quick method for growing the plant (Aguilar et al., 2018). Regenerated embryos (1.25 g) were inoculated in Erlenmeyer flasks (250 mL) with 50 mL liquid growth medium and placed in the dark with constant agitation. After 15 days of incubation, cotyledonary embryos were transferred to a solid medium for plant conversion. Hundreds of high-quality plantlets grew within 8 weeks, without hyperhydration problems (**Figure 2**).

A French group started the use of RITA^®^ temporal immersion bioreactors for the ECS regeneration phases, embryo germination and plant conversion (Berthouly et al., 1995; Etienne et al., 1997a). Subsequently, the germinated embryos were converted into plant by direct sowing into the soil (Etienne-Barry et al., 1999; Barry-Etienne et al., 2002).

#### 2.3.5 Histo-morphological and biochemical characteristics of SE

The development of the coffee somatic embryo is characterized by different histological and morphological stages that include the preglobular, globular, heart, torpedo and cotyledonary phases (Nakamura et al., 1992). Histological *C. arabica* SE development was described by different authors (Söndahl et al., 1979; Michaux-Ferrière et al., 1987; Michaux-Ferrière et al., 1989; Nakamura et al., 1992; Tahara et al., 1995; Menéndez-Yuffá and García de García, 1997). The primary callus proliferates from the spongy mesophyll and perivascular cells of the leaf explant (Michaux-Ferrière et al., 1987; Menéndez-Yuffá and García de García, 1997), but embryogenic cells appear in the periphery of this callus after 20 days of culture, without forming proembryos; some cells become proembryos and then bipolar embryos up to 60 days of culture (Michaux-Ferrière et al., 1989). Embryogenic callus is yellow and friable, with small, isodiametric cells, with dense cytoplasm, prominent nucleus and nucleolus. Non-embryogenic callus is translucent, aqueous, with elongated cells, large vacuoles and without cytoplasmic organelles (Söndahl et al., 1979; Gatica-Arias et al., 2008; Padua et al., 2014; Silva et al., 2014). Embryogenic cells are mitotically active and have high starch (Michaux-Ferrière et al., 1987) and protein (Tahara et al., 1995) contents, whereas proembryos accumulate reserve substances in moderate amounts (Michaux-Ferrière et al., 1987; Michaux-Ferrière et al., 1989). The polarized growth of embryogenic cells occurs after transfer to a hormonally different medium, giving rise to proembryos, which subsequently become somatic embryos (Michaux-Ferrière et al., 1987; Santana-Buzzy et al., 2007). The single-cell origin of coffee SE was observed in somatic embryonic stem cells (Nakamura et al., 1992) and in indirect (Menéndez-Yuffá and García de García, 1997) and direct SE in different *C. arabica* genotypes (Quiroz-Figueroa et al., 2002).

Morphological and histological criteria were evaluated during germination and conversion of somatic embryos in cotyledonary stage and produced in RITA^®^ (Etienne et al., 2013). In the cotyledonary stage, the epidermis of the embryo is well differentiated, however, the embryos lack meristems, the vascular tissue is in formation, as well as the axis of the embryo. After 4 or 6 weeks, the cotyledons showed spongy and palisade mesophyll similar to leaves and vascular tissue was formed. After 10 weeks, a well-formed apical meristem and an axis with two axillary buds were observed. At 12 weeks, the embryos had roots and 60% had a stem and two leaves. It is remarkable that the levels of protein and starch reserves were extremely low throughout the process.

A global analysis of the metabolome and hormonal dynamics was performed using the SE of *C. arabica*, from explant dedifferentiation to embryo development, with the aim of understanding the mechanisms that regulate cell fate and totipotency (Awada et al., 2019). The analysis revealed the existence of five development phases known as “leaf”, “leaf explant dedifferentiation”, “callus”, “redifferentiation” (embryogenic cell clusters to embryoid structures), and the “embryo development” (globular to torpedo-shaped embryos) and four phase changes or transition periods. The dedifferentiation process was characterized by a strong decrease in the levels of phenolic compounds and caffeine; while hormone levels (auxins, cytokinins and ethylene) reached their highest point. In the callus phases, the maximum expression of totipotency was observed, coinciding with the shut-off of the hormonal and metabolic pathways related to the hydrolysis of sugars and reserve substances. The study makes an extensive characterization of the metabolic pathways and the metabolites involved in each phase of development and the transition periods. The accumulation and/or decrease of specific metabolites, including sugars, amino acids, hormones, and chlorogenic acids, is presented. Substances such as abscisic acid, leucine, maltotriose, myo-inositol, proline, zeatin, and tricarboxylic acid cycle metabolites are key metabolic markers of the embryogenic capacity. This knowledge is essential to more accurately understanding and manipulating the mechanisms that regulate the expression of coffee SE.

## 3 Temporary immersion culture: an efficient technology for SE scaling and plant production

### 3.1 Temporary immersion culture in plant biotechnology: A historical background

Micropropagation is carried out commercially only for a limited number of crops. The current technique uses a large number of containers and semi-solid media and considerable labor is involved, increasing the production cost and limiting automation. It has been concluded that commercial application of micropropagation for plant species can only take place if new automated technology is available and if acclimatization protocols are improved (Kitto, 1997).

To overcome the shortcomings of the conventional micropropagation techniques, new methods have been developed using liquid media and bioreactors. The use of liquid media has many advantages for plant micropropagation because they provide more uniform culturing conditions, increase nutrient uptake, promote growth, and reduce plant production costs and are a key factor for automation (Aitken-Christie, 1991). However, the major problems associated with liquid media are asphyxiation and hyperhydricity and, in agitated liquid medium, the shear damage (Etienne and Berthouly, 2002). Temporary immersion reduces these problems; because of the short periods of immersion, asphyxiation does not occur, and because of limited convection of the medium, shear damage is minimized. Teisson and Alvard (1995) described temporary immersion culture as a novel concept for the use of liquid medium in plant tissue culture for the first time.

Since then, a large number of temporary immersion systems (TIS) have been designed; their main differences lie in the type and size of the containers, the method of providing nutrients to the liquid culture (mechanical or pneumatical), and the use of a simple timer or computerized immersion control. Other differences include the recycling of the medium and separation between the plant and medium container. However, the common characteristic of these systems is that containers are larger than conventional culture vessels and have the facility to pass light. Some of them were illustrated and described by Etienne and Berthouly (2002) and grouped by design into four types: systems with rocker machines designed to achieve temporary immersion to combine aeration and the favorable effect of liquid medium culture (Harris and Mason, 1983; Adelberg and Toler, 2004; Robert et al., 2006; Bello-Bello et al., 2010; Peña-Ramírez et al., 2010); systems with complete immersion and a liquid medium renewal mechanism (Tisserat and Vandercook, 1985); systems with partial immersion and with a liquid nutrient renewal process (Aitken-Christie and Davies, 1988; Simonton et al., 1991); and systems with complete immersion of plant material by pneumatic driven transfer of liquid medium and without medium renewal RITA^®^ (Alvard et al., 1993) and Twin- flasks system (BIT^®^) (Escalona et al., 1999). The EBB and FLOW bioreactor (Ziv, 2005) was designed for mass propagation and was operated on the Osmotek LifeReactor System (Product Number 800 554), and its principle was similar to that of RITA^®^ and BIT^®^.

The operational principles and technological design of the most popular TIS have been reviewed by Georgiev et al. (2014). A common feature of all of them is the simple design, low cost utilization, and interchangeable plastics components. The SETIS™ system (Vervit, Karnemelkstraat, Zelzate, Belgium) operates in a similar way as the Ebb and Flow TIS system. PLANTIMA (A-tech Bioscientific Co, Ltd., Huayin St., Zhongshan Dist., Taipei, Taiwan) (Yan et al., 2011) and PLANTFORM bioreactors (Plant Form AB, Hjärup, Skåne, Sweden £ TC propagation Ltd., Ireland) (Welander et al., 2014) operate on the same principle as that of RITA^®^. Box-in-Bag is a disposable TIS, operating on the principle of the Ebb and Flow Temporary Immersion (Ducos et al., 2007c; Ducos et al., 2008). The WAVE bioreactor is a mechanical rocking platform that uses disposable sterilized bags (Eibl and Eibl, 2008). A new 5-L temporary immersion system bigger than RITA^®^ called MATIS^®^ was designed to favor embryo dispersion and light transmittance, enabling the regeneration to *C. arabica* plantlets (Etienne et al., 2013). However, to date, there are no publications demonstrating the successful use of such bioreactor systems for plant micropropagation. Recently, a TIB operated by a microcontroller was designed. The TIB can control the feeding and the CO_2_ concentration (Woowong and Piladaeng, 2022).

A representative summary of type of TIS and its application in different morphogenetic pathways are shown in **Table 3**.

**Table 3:** Representative summary of type of temporary immersion systems and morphogenetic pathway since 2005.

Species	Temporary immersion system	Morphogenetic pathway	References
*Musa* (plantain)	BIT^®^	Shoot proliferation	Roels et al., 2005
*Eucaliptus grandis*	RITA^®^		McAlister et al., 2005
*Alocasia mazonica*	Ebb and Flood		Jo et al., 2008
*Ananas comosus*	BIT^®^		Scherer et al., 2013
*Anoectochilus formasanus* (orchid)	Ebb and Flood		Wu et al., 2007
*Apple* rootstock	RITA^®^		Zhu et al., 2005
*Coffea arabica*	RITA^®^	Somatic embryogenesis	Albarrán et al., 2005
*Dioscorea spp*	BIT^®^	Microtuberization	Jova et al., 2005; Jova et al., 2012
*Tectona grandis*	BIT^®^	Shoot proliferation	Quiala et al., 2012
	RITA^®^		Aguilar et al., 2019
*Discorea fordi* (Chinese yam)	PLANTIMA	Microtuberization	Yan et al., 2011
*Theobroma cacao*	BIT^®^	Somatic embryogenesis	Niemenak et al., 2008
*Camptotheca acuminata*	BIT^®^		Sankar-Thomas et al., 2008
*Solanum tuberosum*	Rocker System	Microtuberization	Kämäräinen-Karppinen et al., 2010
*Vaccinum corymbosum*	BIT^®^	Shoot proliferation	Arencibia et al., 2013
*Peach palm*	BIT^®^	Somatic embryogenesis	Steinmacher et al., 2011
*Coffea canephora*	Box in Bag		Ducos et al., 2010
*Habanero pepper*	BIOMINT	Shoot proliferation	Bello-Bello et al., 2010
*Cedrela odorata*	BIOMINT		Peña-Ramírez et al., 2010
*Saccharum* sp.	RITA^®^		Mordocco et al., 2009
*Carica papaya*	RITA^®^	Somatic embryogenesis	Posada-Pérez et al., 2017
*Coffea arabica L.*	RITA^®^ and MATIS		Etienne et al., 2013
	RITA^®^		Gatica-Arias et al., 2008
			Aguilar et al., 2018
*Coffea canephora*	BIT^®^		Ducos et al., 2007b
*Coffea canephora*	Box- In- Bag		Ducos et al., 2007a; Ducos et al., 2010
*Eurycoma longifolia*	RITA^®^		Mohd et al., 2017
*Phoenix dactylifera*	PLANTFORM	Somatic embryogenesis	Almusawi et al., 2017
*Quercus suber* *Quercus robur*	RITA^®^		Pérez et al., 2013 Mallon et al., 2012
*Chrysanthemum morifolium*	SETIS™	Soot proliferation	Hwang et al., 2022
*Cnidium officinales*			
*Colocasia esculenta*	BIT^®^ Eb-and-Flow SETIS™		Mancilla-Alvarez et al., 2021
*Hylocereus uundatus* (Pitahaya)	Ebb-and-Flow		Bello-Bello et al., 2021
*Gerbera Jamesonii*	BIT^®^	Shoot proliferation	Mosqueda Frómeta et al., 2017
*Bletilla striata*		Pseudobulb induction	Zhang et al., 2018
*Capparis spinosa* L.	PLANTFORM	Shoot proliferation	Gianguzzi et al., 2019
*Anthurium andreanum*	Ebb-and-Flow		Martínez-Estrada et al., 2019
*Vanilla planifolia*	RITA^®^		Spinoso-Castillo et al., 2017
*Camptotheca acuminata*	RITA^®^	Somatic embryogenesis	Sankar-Thomas et al., 2008
*Stevia rebaudiana*	BIT^®^	Shoot proliferation	Vives et al., 2017
*Saccharum Sp* *Sacchararum Sp* *Sacchararum Sp*	SETIS™ BIT^®^ RITA^®^		Da Silva et al., 2020
			Martínez-Estrada et al., 2017
			Meyer et al., 2009
*Dianthus carophyllus L.*	BIT^®^		Ahmadian et al., 2017
*Echinacea purpurea* *Rubus idaeus* *Digitalis Lutea*	PLANTFORM		Welander et al., 2014
*Corema album*	SETIS™		Alves et al., 2021
*Date palm*	PLANTFORM		Abahmane, 2020
*Agave tequilana*	RITA^®^		Vázquez-Martínez et al., 2022
*Lycium barbarum*	PLANTFORM		Ruta et al., 2020
*Bambusa vulgaris*	BIT^®^	Shoot proliferation	García-Ramírez et al., 2014
*Epidendrum fulgens*	PLANTFORM		Fritsche et al., 2022
*Piper aduncum*	RITA^®^	Somatic embyogenesis	De Sousa et al., 2020
*Alnus glutinosa*	RITA^®^PLANTFORM	Shoot proliferation	San José et al., 2020
*Agave tequilana*	BIOMINT	Shoot rooting	Monja-Mio et al., 2020
*Phoenix dactylifera*	PLANTFORM	Somatic embryogenesis	Ali and Welander, 2020
*Agave potatorum*	Ebb-and-Flow	Shoot proliferation	Correa-Hernández et al., 2022
*Colocasia esculenta*	BIT^®^		Arano-Avalos et al., 2020
*Vaccinum vitis-idaea*	BIT^®^		Arigundam et al., 2020
*Musa (AAA)*	SETIS™		Bello-Bello et al., 2019
*Musa (AAB)*	Ebb-and-Flow		Uma et al., 2021
*Solanum tuberosum*	BIT^®^	Microtuberization	Tapia Figueroa et al., 2022
*Orysa sativa*	RITA^®^	Shoot proliferation	Hernández-Soto et al., 2022
*Mimosa calodendrom*	BIT^®^	*In vitro* germination	Souza et al., 2022
*Olea europaea (olivo)*	PLANTFORM	Shoot proliferation	Benelli and De Carlo, 2018
*Saccharum spp*	BIT^®^	Shoot proliferation	Martínez Rivero et al., 2020
*Vanilla planifolia* Jacks	SETIS™	Shoot proliferation	Ramírez-Mosqueda and Bello-Bello, 2021

### 3.2 TIS, a bridge between laboratory and commercial application

In summary, TIS systems developed to date for commercial application are RITA^®^, Twin-Flask system later patented as BIT^®^, Ebb and Flow bioreactor, and SETIS™.

#### 3.2.1 BIT system

**Figure 3:**
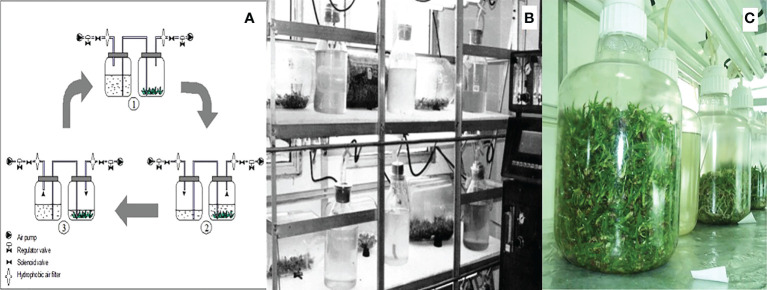
**(A)** Operating cycle of BIT^®^: (1) Non-immersed stage, shoots are free-standing on the bottom of the culture vessel, (2) beginning of the immersed, (3) end of the immersed stage. These steps are performed, e.g., every 3h. The air pump and electric valves were under control of a timer. **(B)** First; **(C)** and second generation of BIT^®^ used to pineapple and sugar cane scaling-up.

BIT^®^ consists of two containers connected by silicone tubes (Escalona et al., 1999). One of the containers acts as a culture chamber, whereas the other is used as a medium storage. Different glass or polycarbonate containers have been used in this system, with sizes ranging from 250 mL to 5 or 10 L. In this system, explants are temporarily immersed by pneumatic driven transfer liquid medium without medium replenishment during one subculture (**Figure 3**). However, in BIT^®^, a new cycle of culture can be started by changing only the medium container with another container with a new nutrient composition and the plant material can be maintained in the culture chamber. To establish a micropropagation procedure and to increase the efficacy of TIB-technology, various parameters should be optimized, including immersion time and frequency, the volume of the nutrient medium and the container (headspace volume container), the type of explant, the duration of the proliferation phase, the use of plant growth retardant, and the number of cycles in BIT^®^.

BIT^®^ has an additional option wherein the headspace can be enriched with CO_2_ during the gas exposure period. Aragón et al. (2010a) described the effects of sucrose, light intensity, and CO_2_ concentration during plantain culture in BIT^®^. CO_2_ enrichment was initiated just 4 h after the light period as follows: during the first week of plant elongation, 6 h; during the second week, 15 h; and during the third week, 24 h per day. The flow was adjusted to 200 mL min^-1^.

The use of additional ventilation, as a factor to improve the efficiency of BIT^®^, was assayed in *in vitro* propagation of *Gerbera jamessoni* (Mosqueda Frómeta et al., 2017). Shoots were ventilated with air filter for 1 min once every 2 h and immersed for 4 min every 6 h over 28 days, and the production of shoots without hyperhidricity was allowed.

The main reason for the efficacy of BIT^®^ is that it allows full contact between explants and medium, as in liquid medium, without asphyxiation. Furthermore, the renovation of the headspace results in O_2_ supply and prevention of CO_2_ and ethylene accumulation (Roels et al., 2006). In addition, the water content is reduced during each immersion period and the hyperhidricity can be controlled.

A successful example of the commercial application of BIT^®^-technology is a scale-up and production facility established at Centro de Bioplantas (Universidad Ciego de Avila, Cuba) for *Ananas comosus* proliferation in a 10-L BIT^®^ (Napoles et al., 2018). To guarantee a safe material for proliferation, the *in vitro* establishment involves microbiological testing against endogenous bacteria and the diagnosis of Wilt virus by molecular tests. Acclimatization and growth are performed in a nursery for six months. In the last two years, with the application of this technology, pineapple varieties have been introduced to companies and farmers over a period of 34 months (“MD-2,” “Perola,” “Champaka,” and Red “Spanish”) to diversify the pineapple production in the country. Massive phenotype observations in the nursery and fields revealed 21.2% of spine plants with “MD-2” and 8% with “Champaka” varieties.

The clonal fidelity of plantain plants produced using BIT^®^ have been also assayed using molecular probes and evaluations conducted in the field at the National Corporation of Bananas in Costa Rica (CORBANA). Data on survival, growth, flowering, harvesting time, fruit production, and SV indicated the efficacy of BIT^®^. In summary, plantain micropropagation gave phenotypes inheritable in the next field generation and DNA content and genetic variabilities were low. In spite of the high genetic stability, a dwarfism-related AFLP-marker is proposed. Phenotype and *in vitro* culture conditions did not affect leaf global DNA methylation. In addition, the use of TIS-metatopolin protocol for plantain micropropagation is advised (Noceda et al., 2012).

**Figure 4:**
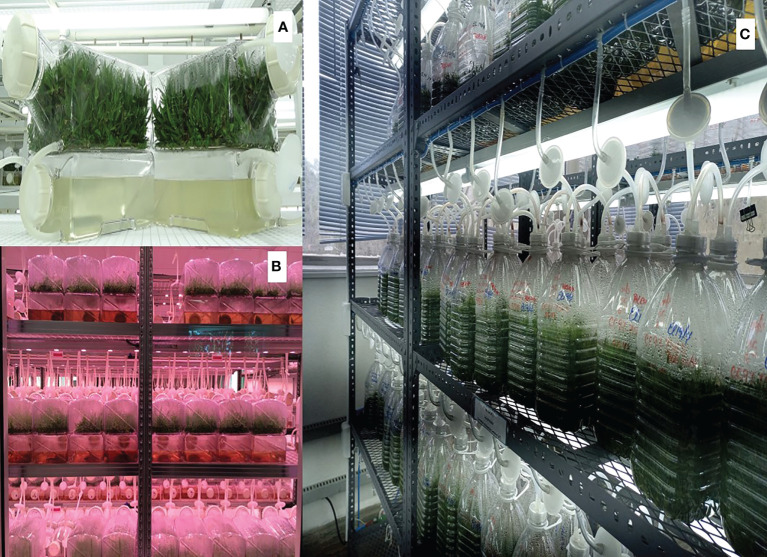
Illustration of the production of sugarcane plantlets using temporary immersion bioreactors. **(A)** SETIS™ bioreactor; **(B)** Florida Crystals Corporation, using SETIS™ technology (https://setis-systems.be/about/about-setis); **(C)** CALESA facilities using BIT^®^ technology.

Furthermore, innovations of the container of 5-L BIT^®^ (water plastic and disposable bottles) were used for the commercial scaling of sugarcane plantlets at Plant Biotechnology CALESA, Panamá. This technology led to a reduction of 10 to 6 years in the introduction of new varieties, and sugarcane plants are produced for basic seeds, commercial uses, and replanting. Currently, 1.5 million plants are produced per year, with a reduction in production costs from 0.45 to 0.12 cents (**Figure 4C**).

#### 3.2.2 SETIS™ system

SETIS™ is a novel concept based on the TIS technology that uses the twin-bottles principle of two connected vessels (plant material and growth media vessels) operating under the ebb-and-flow principle (http://www.Setis-Systems.be. Accessed on 18 june 2022). Most problems and disadvantages of either the commercialized or self-built TIS bioreactors have been solved. Today, TIS has been commercialized and is used by many companies worldwide. SETIS™ has been used for many micropropagation processes with excellent results. For example, PT Tamora Stekindo, Indonesia (Hasfarm Group) have been working on tea multiplication by microcuttings and SE for the last 5 years. There are successful results in callus multiplication and embryo germination. They produce plantlets per SETIS for crops such as bromeliads, sugar cane, banana, alocasia and phalaenopsis. Its standard and optimized design allows ideal plant growth of a wide range of plant species (**Figures 4A, B**).

The efficiency of TIS is unquestionable. TIS provides the most natural environment for *in vitro* culture of shoots, somatic embryos, and micro-tubers. It is recognized as a perspective technology for plant micropropagation, production of secondary metabolites (Pérez-Alonso et al., 2012), transgenic plant selection (Espinosa et al., 2002), and evaluation of an organism’s response to abiotic stresses (drought, salinity, flooding, and high temperature) (Gómez et al., 2019). Another perspective for the application of TIS is the mass propagation of plants used in phytoremediation (Ergönül and Sidal, 2021) or the reduction in the inhibitory effects of phenolics by anaerobic digestion (Kietkwanboot et al., 2022).

#### 3.2.3 RITA^®^ bioreactor

The description and operation of the RITA bioreactor has been widely reported (Teisson and Alvard, 1995; Etienne and Berthouly, 2002). CIRAD was a pioneer in the development of the RITA^®^ bioreactor, whose advantage for micropropagation were clearly demonstrated in the *C. arabica* SE through a high yield and reliable process. It has been the main TIS used for SE pathways of different plant species (**Table 3**). RITA^®^ was intended for mass propagation by SE because it can provide the required conditions for proliferation, maturation, and germination of somatic embryos (Etienne and Berthouly, 2002). Twenty clones of *C. arabica* F1 hybrids were successfully produced using RITA^®^, and the main effect obtained was the improvement in the quality of the resulting embryos. Today, the development of a temporary immersion bioreactor for the mass production of pre-germinated embryos, their direct sowing on soil, and the propagation of rejuvenated somatic embryo plants by rooting mini-cuttings has permitted transfers and disseminations of improved F1 Arabica hybrids and Robusta clones worldwide (Etienne et al., 2018).

#### 3.2.4 Different phases of SE of *C. arabica* using RITA^®^

With the adoption of RITA^®^ bioreactors, *C. arabica* SE was simplified by direct ECS transfer to the bioreactor, enabling regeneration, germination, and conversion in the same vessel (Berthouly et al., 1995; Etienne et al., 1997a), thereby cutting production costs by eliminating the use of solid medium in these phases. In other studies, RITA^®^ bioreactors were used for embryo germination and plant conversion after regeneration on solid medium (Aguilar et al., 2018).

The first embryo regeneration studies of F1 hybrids in RITA^®^ were highly satisfactory (Etienne et al., 1997a), with production rates ranging from 750 to 9000 embryos per container, depending on the hybrid, in 6 months of culture, with high rates of plant conversion (83–95%).The performance in RITA^®^, regarding the production of embryos, was twice higher than in Erlenmeyer flasks (Etienne and Berthouly, 2002).

Controlling the immersion time and frequency, the liquid medium volume, and the amount of inoculum are crucial factors for embryo development in a bioreactor. Germination in RITA^®^ was slower than that in semi-solid medium, but the culture was more synchronized and the material more homogeneous (Etienne et al., 1997a). In RITA^®^, the culture density was assessed based on the quality of germinated embryos. At a high density (1600 embryos), embryo growth was superior after 3 months in the greenhouse (Etienne-Barry et al., 1999). The initial volume was adjusted to 200 mg of embryogenic cell clusters and 200 mL of liquid medium. Subcultures were performed every 2 months, and the embryos reached the cotyledonary stage after 4 months. At the end of the development phase, the bioreactor content was divided into different containers until an optimal density (700 embryos) was reached for germination (Etienne-Barry et al., 1999; Barry-Etienne et al., 2002; Albarrán et al., 2005).

Varying the immersion time affects embryo production. Frequent immersions (every 6 h) for a long time (15 min) allowed embryo development and germination, but a strong reduction in this parameter (daily 1-min immersion) blocked embryo development and favored secondary embryogenesis (Teisson and Alvard, 1995). Albarrán et al. (2005) evaluated 1-min immersions every 24, 12, and 4 h and found high-quality embryo production (60%, 79%, and 85%) proportional to the increase in immersion frequency. Two daily 1-min immersions is the commonly used immersion frequency for *C. arabica* (Etienne-Barry et al., 1999; Barry-Etienne et al., 2002; Menéndez-Yuffá et al., 2010; Etienne et al., 2013). When 1-, 5- and 10-min immersions every 8 h were evaluated in cv. “Catuaí”, short 1-min immersions resulted in the best embryo development (Gatica-Arias et al., 2008).

**Figure 5:**
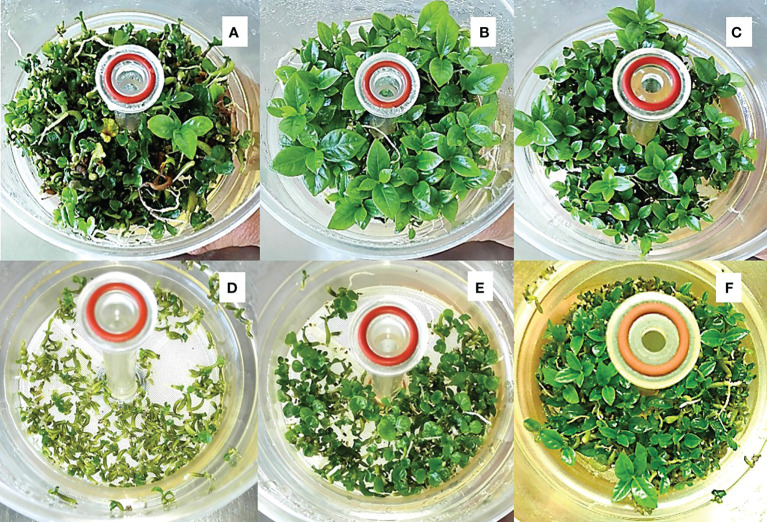
Germination of somatic embryos and conversion into plants in liquid T5 culture medium in RITA^®^ bioreactors. **(A)** Control culture, with a frequency of two daily immersions of one minute; observe the asynchrony in the development and hyperhydricity of the culture; **(B)** good quality plants cultured using 2 one-minute immersions every 48 h; **(C)** plants cultured using 1 immersion every 48 h; **(D)** green embryos after the first month of culture in RITA^®^; **(E)** embryos in the cotyledon stage in the second month of culture in RITA^®^; **(F)** plants with one or two pairs of leaves in the third month of culture in RITA^®^.

In another study of F1 hybrids, at an initial density of 1.25 g of regenerated embryos, 1 or 2 daily 1-min immersions led to a high number of malformed, hyperhydric, and dead embryos (Aguilar et al., 2017). When 2 daily 1-min immersions (control) with 1 or 2 1-min immersions every 48 h were compared, the number of embryos decreased but synchronization and plant conversion improved (**Figures 5A–C**). The average number of high-quality plants (74) per sample (10 g) was better after 2 1-min immersions every 48 h than after 2 daily 1-min immersions (12).

**Figures 5D–F**, shows embryo germination and plant conversion during 3 months in RITA^®^, when the growth medium was replaced monthly (Aguilar et al., 2018). In the first month, the embryos elongated and the cotyledons developed and turned green (**Figure 5D**). In the second month, many embryos reached the cotyledonary stage, some formed roots, and others remained in early stages (**Figure 5E**). Given the increase in biomass, some containers were divided in the second or third month to reduce density and favor germination. In the third month, many embryos developed into plantlets with one or two pairs of true leaves and roots (**Figure 5F**). From this period to the fifth month, the plantlets were harvested for acclimatization.

#### 3.2.5 Problems associated with temporary immersion culture

Despite favoring nutrient uptake and promoting growth, culture in liquid medium has limitations, such as suffocation and hyperhydricity (Etienne and Berthouly, 2002). Hyperhydricity results from stress-inducing *in vitro* culture conditions (Kevers et al., 2004) and could account for poor growth and numerous losses during and after cultivation (Etienne et al., 2006). Hyperhydricity is a physiological disorder induced by physical and chemical factors (Ivanova and Van Staden, 2009; Ivanova and Van Staden, 2011) such as high relative humidity (McAlister et al., 2005; Troch et al., 2010; Akdemir et al., 2014; Vidal et al., 2015), high PGR concentrations (Kevers et al., 2004; Bairu et al., 2007; Moncalean et al., 2009; Quiala et al., 2012), gas accumulation, light intensity, and temperature (Saher et al., 2004; Wu et al., 2011).

Biochemical characteristics are associated with morphological abnormalities of the hyperhydric state (Gaspar, 1991; Kevers et al., 2004); reduced dry mass; high water content; and low cellulose, chlorophyll, and lignin contents (Kevers et al., 1984). Consequently, hyperhydric plants are turgid, watery, and hypolignified, translucent, and less green. Other features include increased enzymatic activity (glutamate dehydrogenase and basic peroxidases) related to amino acid synthesis and auxin catabolism (Kevers et al., 2004).

Morphologically, hyperhydric plants have wide stems, short internodes, and thick, elongated, wrinkled and brittle leaves (Kevers et al., 2004) due to marked anatomical alterations (Gao et al., 2018), such as hypertrophy of the cortical parenchyma, atypical vascular bundles, and little xylem lignification. In leaves, the palisade mesophyll is reduced or absent, and the intercellular spaces are large (Kevers et al., 2004), with an irregular epidermis and cuticle and low stomatal density (Gao et al., 2018). The stomata lack a closing mechanism, which could cause accelerated water loss and increased vulnerability during acclimatization (Kevers et al., 2004).

Temporary immersion bioreactors promote temporary contact between plants and the liquid medium (Alvard et al., 1993; Escalona et al., 2003a). This contact is regulated by immersion cycles that control water and nutrient absorption, reducing or avoiding hyperhydricity (Etienne and Berthouly, 2002; Albarrán et al., 2005; Etienne et al., 2006). In *C. arabica*, hyperhydricity mainly occurs during germination and plant conversion, favored by prolonged immersions and not so much by frequency, under short immersion periods (Etienne and Berthouly, 2002). In addition, 15-min immersions every 4 and 12 h increased hyperhydricity by 90 and 64%, respectively. The highest germination and plant conversion rate (75%) was after 1-min immersions every 4 h (Albarrán et al., 2005). Aguilar et al. (2017) observed that the frequency of immersion affected the hyperhydricity of the culture (**Figure 5A**), a condition that was halved by decreasing the frequency of 1-min immersions from 12 to 48 h (**Figures 5B, C**).

In woody species such as eucalyptus (McAlister et al., 2005), apple (Zhu et al., 2005), chestnut (Troch et al., 2010; Vidal et al., 2015), pistachio (Akdemir et al., 2014), and teak (Quiala et al., 2012; Aguilar et al., 2019), the frequency of immersion affected the hyperhydricity. This factor is crucial for nutrient uptake, multiplication rate and hyperhydricity control because explants come into contact with the nutrient medium in each immersion, and growth and multiplication possibly occur during the interval between immersions (Ahmadian et al., 2017). In eucalyptus, the increase in the frequency of immersion increased the multiplication rate and plant quality (McAlister et al., 2005). In teak, a 1-min immersion every 48h reduced hyperhydricity (7%) and improved shoot quality and acclimatization survival (52%) (Aguilar et al., 2019).

The hydric parameters are strongly affected by the immersion cycles and directly affect hyperhydricity (Etienne and Berthouly, 2002). Hyperhydric coffee embryos had higher fresh weight and water content and a more negative water potential than normal embryos, in addition to structural problems and inability to germinate (Albarrán et al., 2005). The high fresh weight (124.6 mg) of regenerated cv. “Catuaí” plants, in contrast to the low plant conversion (45.3%), could be related to hyperhydric or malformed embryos. Although germination rate was high (100%), not all embryos were viable (Gatica-Arias et al., 2008).

Structurally and physiologically abnormal plant development is caused by growth conditions that favor rapid growth and multiplication (Hazarika, 2006). High cytokinin concentration and inoculum density and prolonged culture time promote hyperhydricity and abnormal plant development (McAlister et al., 2005; Zhu et al., 2005; Troch et al., 2010; Akdemir et al., 2014), both on solid medium (Moncalean et al., 2009; Aguilar et al., 2019) and in temporal immersion (Zhu et al., 2005; Quiala et al., 2012; Vidal et al., 2015; Aguilar et al., 2019). However, in kiwi, the incubation time in BA (6-Benzylaminopurine) had a stronger effect on plant quality than the concentration, affecting the water content and holding capacity (Moncalean et al., 2009). In teak, cultures in BIT^®^ (Quiala et al., 2012) and in RITA^®^ (Aguilar et al., 2019) showed that high BA concentrations increase not only the number of shoots but also the hyperhydricity and are correlated with a low content of total phenols and shoot hypolignification (Quiala et al., 2012). BA affects the metabolism of phenols, particularly lignin or its precursors, and weakens the vascular cell wall, decreasing its hydrophobicity and water permeability, thereby causing hyperhydricity (Hazarika, 2006).

Culture time and high and low inoculum densities affect the morphological and physiological development of plants in temporary immersion (McAlister et al., 2005; Vidal et al., 2015; Aguilar et al., 2019). Both high and low *C. arabica* embryo densities in RITA^®^ favored germination and plant conversion during direct sowing in the greenhouse. Although the authors did not evaluate hyperhydricity, they mentioned that high density causes physical restrictions in embryos, affecting their morphology during germination (Etienne-Barry et al., 1999).

Hyperhydricity is reported as an important limitation of somatic embryo cultures in TIB (Etienne et al., 2006; Egertsdotter et al., 2019; Vidal and Sánchez, 2019). However, most studies on SE of woody species in temporary immersion fail to address this problem.

Asynchronous development and embryo malformation are common features observed during SE and culture in RITA^®^. Embryos with multiple or no cotyledons, multiple or absent rudimentary axes (Ammirato, 1989), and nonfunctional or absent apical meristems (Dodeman et al., 1997) are also observed in coffee. In the same RITA^®^, embryos can be found at different stages of development, and plant conversion is asynchronous, thereby requiring 2 or 3 harvests (Etienne et al., 2012). Embryo subcultures in two or more RITA^®^ TIS is a practice used for coffee to lower the density and favor crop synchronization, embryo development, and plant conversion of the most developed embryos. **Figures 5D-F** shows the increase in embryo density during three months of culture in RITA^®^. At the third month (**Figure 5F** **)** plantlets are observed on the surface of the container, and embryos in earlier stages in the lower part. This is the time to divide the RITA^®^ (Aguilar et al., 2018). The bioreactor RITA^®^ improved *Citrus deliciosa* SE synchronization by hindering secondary embryogenesis at the start of germination (Cabasson et al., 1997); in *Quercus robur*, synchronization improved after 1-min immersions every 48 h, increasing the production of cotyledonary embryos (90%) (Mallon et al., 2012); and the same immersion cycle in *C. arabica* (**Figure 5C** **)** improved synchronization and embryo quality (Aguilar et al., 2017).

In the bioreactor, embryo heterogeneity induces variability in plant conversion efficiency in soil and in delays growth in the nursery (Barry-Etienne et al., 2002). The success of temporary immersion culture depends on the optimization of the factors that affect the culture environment of each container and each biological system.

#### 3.2.6 Physiological, biochemical, and molecular aspects of plant response to temporary immersion

Considerable expertise and interpretation is available on the physiology process in semi-solid medium; however, a definition of the physiological requirement of explants and eco-physiological characterization of the bioreactor environment is required to facilitate an increase in plant quality. Knowledge on the effect of temporary immersion culture on plant physiology is essential to optimize culture conditions when a bioreactor is used.

A study on the effect of BIT^®^ on the physiology of micropropagated pineapple plantlets was conducted by Escalona et al. (2003b). CO_2_ concentration in the headspace measured during an entire cycle of immersion increased 6.9-fold in comparison with that in the semi-solid culture. The accumulation of CO_2_ was a consequence of plant metabolism rather than an environmental response as a consequence of higher respiratory activity of proliferated shoots, but this fact did not seem to affect the intrinsic quality of the shoots. The low photosynthetic capacity induced by BIT^®^ was consistent with the large increase in sugar and nitrogen uptake and in the dry weight and foliar area exhibited by these plantlets. The authors agree that whether or not plantlets are photosynthetically active at transplantation is of secondary importance.

The composition of the headspace during proliferation and prior to acclimatization of plantain shoots in BIT^®^ and semi-solid culture showed large differences in gas concentrations. There was a higher CO_2_ and a lower O_2_ peak during the elongation phase compared to that in the proliferation phase. The renewal of the headspace, with every immersion, resulted in a supply of O_2_ and prevention of CO_2_ and ethylene accumulation, which had a favorable influence on the quality of plantain shoots (Roels et al., 2006).

The timing of photosynthesis and changes in the sucrose content, as well as the activities of some key enzymes involved in carbon metabolism, were described during *in vitro* culture of plantain shoots in BIT^®^ and during acclimatization (Aragón et al., 2005). In the *in vitro* stage, photosynthesis was limited (4−6 µmol CO_2_ m^-2^s^-1^); the photosynthetic rate, however, increased rapidly and significantly as soon as *in vitro* acclimatization was over. Starch was the most important sugar accumulated by the stems. Plantain plantlets begin starch uptake during the first days of acclimatization. This accumulation, as a nutrient reserve, is important to overcome the stress period during the subsequent growth under *ex vitro* conditions. The growth parameters of plantain shoots before acclimatization indicate that the system improves the plant quality compared with the semi-solid culture.

To identify the cellular features that support the quality of BIT^®^ plantlets at the levels of anti-oxidative stress, *in situ* reactive oxygen species (ROS) deposition, the activity of anti-oxidative enzymes, and the expression of the corresponding genes were compared in *ex vitro* plantain plantlets propagated *in vitro* through semi-solid and BIT^®^ culture (Aragón et al., 2010b). This study elucidated the mechanisms by which BIT^®^ grown plantlets can better sustain and overcome oxidative stress than semi-solid grown plantlets, enhancing their propagation capacity and improving their growth.

Similar results in sugarcane showed that plantlets propagated in BIT^®^ had better morphology and physiological behavior than plantlets propagated in semi-solid culture (Aragón et al., 2009). The evidence suggests that BIT^®^ propagated plantlets, at the moment of transfer to *ex vitro* conditions, had functional stomata and trichomes with characteristics similar to those of *ex vitro* plants. BIT^®^ plantlets maintained the anti-oxidative system activated from the *in vitro* phase, and the major ROS scavenger, super oxide dismutase (SOD), was present at high levels of activity until the end of acclimatization. In the mesophyll and bundle sheath cells and within the different cell compartments, H_2_O_2_ produced by SOD activity was processed by the glutathione reductase branch of the ascorbate-glutathione cycle. The polymerization of peroxiredoxins was, apparently, a transient solution for the oxidative environment in chloroplast and mitochondria. All these data suggest the better preparation of BIT^®^ plantlets to cope with the stress of acclimatization and the adjustment of the autotrophic behavior to the antioxidative response to *ex vitro* environment.

The BioMINT II™ bioreactor was used to evaluate the efficiency of this system compared to semi-solid culture during sugarcane micropropagation. BioMINT II™ was evaluated in terms of biomass production and morphological and physiological parameters. Furthermore, RT-qPCR was performed to analyze the expression of two genes (P1P2;1 and EIN3) involved in plant development as possible molecular markers. At day 28, more shoots and better-quality physiological parameters were obtained in BioMINT II™ than in the semi solid culture. The expression of both genes in correlation with morphological parameters suggested the expression of PIP2;1 as an indicator of root development and the expression of EIN3 as an indicator of leaf and root development (Carrillo-Bermejo et al., 2019).

The success of temporary immersion culture not only depends on obtaining a greater number of plants, but also improves the morphological and physiological indicators that guarantee a greater survival of the plants in *ex vitro* conditions.

The positive effect of TIB culture on SE has been evidenced in many woody species, such as *Hevea brasiliensis* (Etienne et al., 1997b), *Psidium guajava* (Kosky et al., 2005), *Theobroma cacao* (Niemenak et al., 2008), *Quercus robur* (Mallon et al., 2012), *Bactris gasipaes* (Heringer et al., 2014), *Elaeis guineensis* (Gomes et al., 2016), *Carica papaya* (Posada-Pérez et al., 2017), and *Larix × eurolepis* (Le et al., 2021). However, in most of these species, the technique is still at an experimental level.

In *H. brasiliensis*, temporary immersion was used to study the induction of oxidative stress on an embryogenic callus during the immersion stage (Martre et al., 2001). A 1-min immersion was enough to cause an increase in SOD activity and high lipid peroxidation, which disappeared in less than 1 h after the end of the immersion stage.

In *B. gasipae* Kunth, the use of RITA^®^ improved the efficiency of the SE protocol (Heringer et al., 2014). The study also evaluated global DNA methylation levels, total protein and starch contents, and alcohol dehydrogenase activity (ADH). The RITA^®^ showed enhanced multiplication of somatic embryos with increased protein and starch contents and ADH activity. A low DNA global methylation rate (27.52%) was observed, suggesting its relationship with the expression of proteins associated with the maturation of somatic embryos and their subsequent conversion to plantlets. These results showed the importance of defining morphological and physiological features related to the morphogenetic process in peach palm.

Regeneration of somatic embryos in *T. cacao* L. in BIT^®^ proved to be suitable for mass production of torpedo-shaped somatic embryos. Matured embryos pre-treated with 6% sucrose were converted into plants after direct sowing. Additionally, the influence of culture conditions on the content and composition of amino acids was analyzed. The content of free amino acids in somatic embryos increased as the immersion frequency increased. The endogenous free GABA content in embryogenic callus was significantly higher than that in non-embryogenic callus (Niemenak et al., 2008).

The most spectacular effect obtained by temporary immersion was a dramatic improvement in the quality of the resulting coffee somatic embryos, which was 90% that of normal torpedo-shape embryos, and this quality was also reflected in high levels of plant conversion (80−90%) in semi-solid culture (Etienne et al., 2018). Currently, the use of RITA^®^ for the mass production of pre-germinated embryos, their sowing on horticultural soil (Etienne-Barry et al., 1999), and the propagation of the rejuvenated plantlets from somatic embryos by rooted mini-cutting (Georget et al., 2017) has been a decisive innovation for successful scaling-up and reduction of the production cost to disseminate improved F1 Arabica hybrids and Robusta clones (Etienne et al., 2018). However, scientific information on the physiological changes related to the pre-germinated process in a temporary immersion bioreactor is lacking. This study focusses on the morphological aspects. The authors clearly demonstrated that the heterogeneity of the embryos in the bioreactor resulted in variability in both the plant conversion efficiency in soil and plant growth in the nursery. However, scientific information on the physiological changes related to the pre-germination process in a temporary immersion bioreactor is lacking.

In *C. arabica*, the age of SE plantlets harvested from the nursery is an important factor for successful rooting and growth (Campos et al., 2017). This calls for detailed physiological studies; the knowledge of the main physiological changes during this process and the possibility to manipulate these factors can improve the transition from heterotrophic to autotrophic metabolism.

Furthermore, it is important to obtain more knowledge regarding the effect of the composition of headspace in the pre-germination of embryos in the bioreactor. Various studies reported the importance of aeration in coffee somatic embryos. A mixture of air enriched with 5% CO_2_ led to more embryos than the control without CO_2_ enrichment (Barbón et al., 2008). It was observed in Robusta clones that the microenvironment stimulates the *ex vitro* germination rate, and this positive effect is linked to the release of CO_2_ by commercial horticultural substrates (Ducos et al., 2009). To date, attempts to stimulate the photoautotrophic transition in *C. arabica* using a high concentration of CO_2_ combined with high irradiation are being made. Furthermore, the rates of success in the conversion of embryos into plantlets have been improved (Etienne et al., 2018).

Coffee SE is one of the most advanced technologies. It has been industrialized (Robusta) or commercialized (Arabica) for 13−15 years for the two cultivated species. Very high yields and biological efficiencies characterize both processes, with low genotypic effects and controlled SV (Etienne et al., 2018). However, losses during their acclimatization in nursery increase production cost. Therefore, better knowledge of the physiology of transit from *in vitro* to *ex vitro* conditions could minimize and control this loss.

## 4 Production of *C. arabica* plants *via* SE

### 4.1 Acclimatization and *ex vitro* plant production

**Figure 6:**
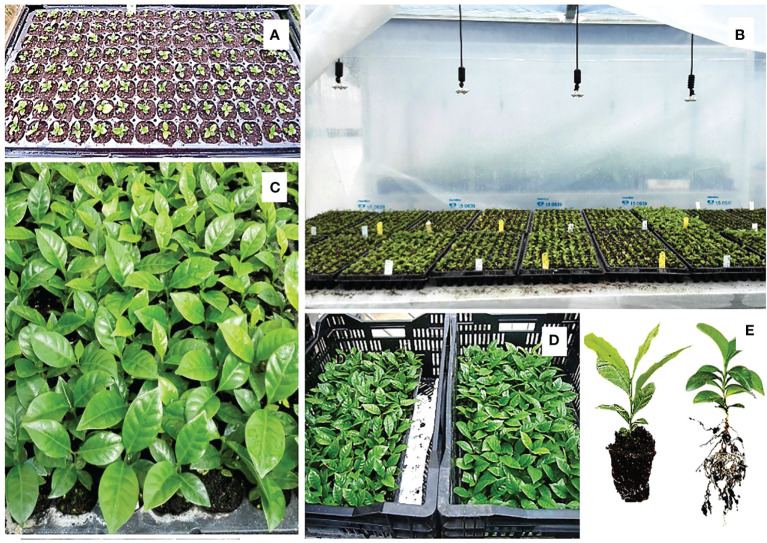
Acclimatization of coffee plants produced by somatic embryogenesis. **(A)** Plants with at least one pair of leaves planted in a tray; **(B)** plastic tunnels for acclimatization; **(C)** plants acclimated after 4 months in the greenhouse; **(D)** mother plants ready for transfer to nurseries for horticultural propagation; **(E)** morphology of an acclimatized mother plant; observe the good root system.

Embryos were converted into plants both in a bioreactor (Etienne et al., 1997a; Aguilar et al., 2018) and directly on horticultural soil (Etienne-Barry et al., 1999; Barry-Etienne et al., 2002). Plastic tunnels containing an automated irrigation system were traditionally used to acclimatize plantlets with 3 or 4 pairs of true leaves (Etienne et al., 1997a). **Figure 6** shows the acclimatization of F1 hybrids using plantlets with at least one pair of true leaves, with acclimatization percentages ranging from 45 to 85%, depending on the genotype (Aguilar et al., 2018).

Direct sowing of the germinated embryos obtained *via* RITA^®^ in horticultural soil (Etienne-Barry et al., 1999) simplified SE and lowered production costs by reducing the handling time (13%) and shelf space (6.3%) compared with conventional acclimatization (Barry-Etienne et al., 2002). Initially, this method provided 40% acclimatization (Etienne-Barry et al., 1999). Embryos with cotyledons of intermediate size showed improved *ex vitro* conversion rate (63%), and the plantlets had a better organized anatomical structure than plantlets germinated *in vitro* (Barry-Etienne et al., 2002), possibly indicating an enhanced adaptation to the *ex vitro* environment. Direct sowing of germinated embryos in a bioreactor was also reported by Aguilar et al. (2018) with a more than 40% success rate; however, many embryos remained in that state and never developed.

**Figure 7:**
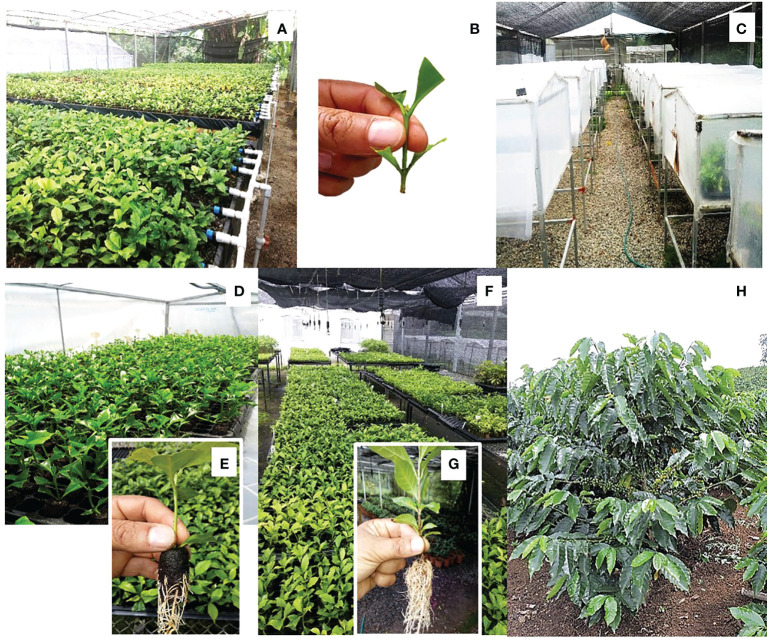
Clonal propagation of coffee mother plants from rooted cuttings. **(A)** Hydroponic garden with mother plants from SE; **(B)** prepared cutting 7 cm long, with two nodes and four pruned leaves; **(C)** exterior of the tunnels for rooting cuttings; **(D)** interior of the tunnels for rooting; **(E)** cutting rooted in ‘Jiffy^®^’ pellets after 40 days in the rooting tunnels; **(F)** greenhouse for acclimatization of newly rooted plants before their transfer to the nursery; **(G)** root system of a rooted cutting; **(H)** plant derived from a rooted cutting, 1 year of age, CATIE, Costa Rica.

Using mother plant clones (**Figure 7**), mini-cuttings were rooted in a greenhouse (Mesén and Jiménez, 2016) at high densities in hydroponic beds for continuous and prolonged sprouting (**Figure 7A** **)**. The sprouts were harvested every 15 days, and production was maintained for more than three years. Leaf cuttings with two nodes (**Figure 7B**) were treated with commercial auxins and added to plastic tunnels for rooting (**Figure 7C**). Under a relative humidity of more than 80% and a daytime temperature of 30−35 °C, the rooting time ranged from 7 to 8 weeks (**Figures 7D, E**). Subsequently, the rooted plants were acclimatized in the tunnels without curtains, and the plantlets remained under those conditions for a week (**Figure 7F**) before being transferred to a common greenhouse (**Figure 7G**) with 60% shade and watering more than one week apart (Aguilar et al., 2018).

**Figure 8:**
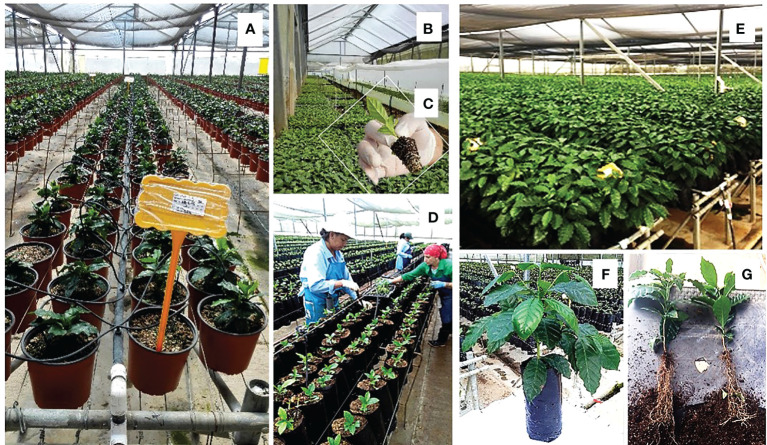
Industrial multiplication of coffee F1 hybrids. **(A)** Cutting producer mother plants at the Paraiso-Gaia Artisan Coffee farm (Costa Rica); **(B)** plastic tunnels for rooting cuttings; **(C)** rooted cutting; **(D)** planting of rooted cuttings in bags; **(E)** plants developed over 5 months at Gaia Artisan Coffee; **(F)** aerial development of plants in bags; **(G)** development of the root system in 5-month old plants.

The strategic alliance with companies specialized in large-scale vegetative propagation enables the mass production of F1 hybrids from rooted cuttings (**Figure 8**). Five months was the average time for a plant to develop from rooted cuttings, in a bag, with a balance between shoots and roots and with the plant being commercially acceptable. Under adequate health conditions throughout the process, the mother plants can produce plants at a rate of 1,900 cuttings per m^2^ per year for 24 months. Thus, the costs of the *in vitro* material are reduced, and a higher number of plants are produced (Aguilar et al., 2018).

### 4.2 Somaclonal variation during SE of *C. arabica*

The production of off-type plants (SV) is another limitation of SE that can result in a drastic phenotypic variation due to genetic and epigenetic variations (Correia et al., 2016). Therefore, research on coffee has focused on mitigating the impact of SV and facilitating early molecular detection or on the efficient selection of variants in the greenhouse (Etienne et al., 2016). *C. arabica* SV was first reported in 1992 by Söndhal et al. with a frequency ranging from 3 to 30%, depending on genotype and protocol (Ducos et al., 2003).

HFSE is associated with genetic or epigenetic instability inducers, such as 2,4-D and ECS; thus, different approaches have made it possible to assess the effect of cultivation techniques on SV in Arabica coffee. Based on morphological and agronomic criteria (Etienne and Bertrand, 2001; Etienne and Bertrand, 2003; Menéndez-Yuffá et al., 2010), seven phenotypic variants were identified, along with the effects of the genotype and suspension age on SV frequency (Etienne and Bertrand, 2003). These variants are known as “Juvenile leaf color,” “Giant,” “Dwarf,” “Thick leaf/Bullata,” “Variegata,” “Angustifolia,” and “Multi-stem.” All variants except for “Multi-stem” have been reported in seed plants. Variant plants are characterized by low productivity and reduced vigor. In most genotypes, SV reached 1.3% in plants from 3-month ECS; however, suspension age and genotype had a strong impact on variant severity and frequency (Etienne and Bertrand, 2003). The impact of culture time and PGR concentration was analyzed by Bobadilla Landey et al. (2013; 2015), who observed that low 2,4-D concentrations (0−1.4 µM) during 6 months of cultivation induced low variation rates (0.74%), while high 2,4-D (4.5 µM) and 6-BA (17.8 µM) concentrations during 11 and 27 months of cultivation generated 30−94% SV; further, plants derived from 4-month cultures were normal.

Amplified fragment length polymorphism (AFLP) markers were studied in *C. arabica* plants regenerated by direct and indirect SE (Santana-Buzzy et al., 2007). Of the 1,446 bands analyzed, 11.4% were polymorphic and 84% were specific to plants regenerated in both processes and in mother plants. Nevertheless, in F1 hybrids, very low polymorphisms were observed with AFLP and methylation-sensitive amplified polymorphism markers in mother plants (0−0.003%), with respect to ECS and secondary embryogenic plants (0.07−0.18%), in line with the low SV rate (0.74%) observed in 200,000 plants in the field. Furthermore, very low polymorphic methylation profiles (0.087–0.149%) were observed regardless of culture age (Bobadilla Landey et al., 2015). The chromosome count reflected the loss of 1−3 chromosomes in some variants (Bobadilla Landey et al., 2013), indicating that mitotic aberrations play a key role in coffee SV (Bobadilla Landey et al., 2015; Etienne et al., 2016; Campos et al., 2017). In summary, *C. arabica* SE based on ECS culture is efficient and safe for the reliable propagation of selected materials because more than 99% regenerated plants are morphologically consistent, with limited genetic and epigenetic changes (Etienne et al., 2016).

## 5 Conclusions: Challenges and perspectives of SE of *C. arabica* in temporary immersion culture

Recent innovations in the industrial production of elite plants of different generations of *C. arabica* F1 hybrids through SE have been presented in detail by a French research group (Etienne et al., 2018). Their synopsis of the progress achieved from the pilot scale to commercial production spanned from 1995 to 2018. The scope of this technology is highlighted in the successful transfer to different countries in Latin America, Africa, and Asia. Despite limitations in the widespread distribution of these materials, including the high cost of plants, lack of capital of smallholder farmers, variations in the price of coffee, and difficulties accessing smallholdings, they mention that 20 million hybrid plants were distributed in Central America in the last decade.

Unsolved technical factors of SE, together with logistical problems and the lack of promotion and funding policies, have limited the distribution of F1 hybrids in Central America for more than a decade (Aguilar et al., 2017). In an additional effort to transfer elite materials to national producers, CATIE in Costa Rica uses a three-phase strategy for the multiplication of F1 hybrids: 1) hybrid regeneration by SE; 2) the establishment of clonal gardens in greenhouses for rooting mini-cuttings to quickly multiply plants from SE (Mesén and Jiménez, 2016); and 3) strategic alliance with companies specialized in industrial cloning, which was a key step for the mass production of F1 hybrids and for the transfer of plants to producers at a lower cost than that of plants directly sourced from the laboratory. The Costa Rican company Gaia Artesan Coffee estimated that the cost of a finished plant for plantation could be lower than US$0.75 (Aguilar et al., 2018).

However, considering the importance of Arabica coffee in Central America, where the economy of these countries revolves around coffee production, this activity must be strengthened with more productive varieties, with higher cup quality to meet the current demands, with resistance to diseases and pests, and with characteristics favoring adaptability to climate change challenges. Although F1 hybrids have been selected for some of these benefits and have been available for more than a decade, they have not been distributed to farmers in the required quantities and at competitive prices due to the complexity of propagation technology and lack of investment (Aguilar et al., 2018). In Central America, effective access to coffee hybrids has been limited to medium- and large-scale farmers, being prohibitive for smallholder farmers given the high cost and absence of distribution channels. These materials have been transferred to smallholder producers through donations from agronomic research institutions (Etienne et al., 2018).

New generations of hybrids, resulting from genetic improvement programs, with new characteristics and appropriate for the current needs of elite materials, are being released (https://worldcoffeeresearch.org/work/annual-report-2016). Therefore, efficient vegetative propagation techniques will be required for their cloning.

Although *C. arabica* SE is indeed a successful example with remarkable results in the production of genetically stable plants, in a large number of genotypes, technical and methodological adjustments are necessary at different stages of the process to reduce outgrades. In addition, knowledge on the fundamental aspects of coffee SE must be deepened using modern tools for advancing research in a more technical and less empirical manner. Improvements in some phases of coffee SE are necessary for this tool to be applicable to a greater number of genotypes. Improving EC production and establishing cryogenic cultures of these calluses and ECS are necessary for storing and managing embryogenic material, especially for the most recalcitrant genotypes.

Although the use of bioreactors reduces costs, the initial outlay is high, some parts must be replaced after several autoclave cycles, and solutions often must be improvised (Vidal and Sánchez, 2019). The existing technology must be improved using low-cost automated systems and containers to control the parameters that affect the efficiency of these systems and to reduce losses due to low-quality plants. Hyperhydricity, the inability to establish polarity during proliferation, and the availability of light as a critical factor during germination are factors that should be improved in current bioreactors (Vidal and Sánchez, 2019). Increasing plant conversion rates and acclimatization survival is another unaddressed challenge (Aguilar et al., 2018).

Further knowledge on the physiological changes during different phases of bioreactor cultivation must be obtained to scientifically explain the frequent morphological variability observed in cultures. Hyperhydricity, asynchronous development, morphological variability of embryos during pre-germination, and their negative impact on plant conversion and growth in the nursery could be explained and improved physiologically. Further information on the physiological metabolism of the transition phase from the heterotrophic (*in vitro*) to the autotrophic (*ex vitro*) environment could allow the modulation of these factors and reduce the impact of plant loss in nursery and production costs.

The information generated by omics technology will have a profound impact on the understanding of the molecular mechanisms related to SE. Analysis of data on the genome, transcriptome, proteome, and metabolome will help identify the regulatory mechanisms responsible for reprogramming gene expression and provide basic information regarding the dedifferentiation process underlying SE (Pais, 2019). For example, knowledge on genes encoding transcription factors provides information on the regulation of SE induction (Karami et al., 2009; Guan et al., 2016). In addition, embryogenic competence markers can be obtained from transcriptome and proteome data, thereby enabling a more precise selection of explants and increasing the reliability of SE. However, one of the main challenges is to identify the changes in the proteome of a somatic cell that trigger all phases of development of an embryogenic cell until the germination of the somatic embryo (Aguilar-Hernández and Loyola-Vargas, 2018). Furthermore, global metabolite analysis could help predict all cell-fate transitions, from leaf explant introduction to embryo development, and could be used to monitor the key stages of coffee SE from a scientific rather than an empirical perspective, as conducted thus far (Awada et al., 2019).

The implementation of shared propagation systems between partners or institutions is a good strategy for simplifying the plant production chain, socializing and transferring technology, and lowering production costs. The multiplication of coffee hybrids by combining *in vitro* technologies with classic high-yield vegetative propagation strategies will make it possible to meet the demand for elite materials for planting, in addition to reducing costs per plant according to the increase in demand. The results, both at the experimental level and in commercial farms, indicate that rooting coffee cuttings using juvenile mother plants of SE is a reliable way for multiplying plants on a large scale and in a short time, with a simple and low-cost technology (Mesén and Jiménez, 2016; Georget et al., 2017).

Although this technology reduces the cost of the plant for the farmer, other challenges in the production chain must also be addressed. Farmers need support with effective financial strategies to invest in the renewal of coffee plantations with quality genetic material to face climate change vulnerability and to meet increasingly stricter market demands. Therefore, an effective link between the research and innovation sectors and the public and private governing institutions of the coffee sector is necessary, both to generate the appropriate technology and to establish effective communication channels that facilitate technology transfer. Technical monitoring by coffee-governing institutions is essential to guide producers on the technical rigors, advantages, and challenges of growing high-productivity hybrid materials. Similarly, training producers on the advantages and added value of growing elite materials will create confidence in investing in the adoption of these materials.

## Data availability statement

The original contributions presented in the study are included in the article/supplementary material. Further inquiries can be directed to the corresponding author.

## Author contributions

MEA and X-yW conceived the article. MEA organized the work plan, wrote everything related to somatic embryogenesis, introduction, conclusions and integrated all the topics. ME wrote the temporary immersion culture chapter and assisted in revising the entire manuscript. X-yW, LY, and LH provided support with information on the chapter on somatic embryogenesis and the aspects on Robusta coffee. All authors contributed to the article and approved the submitted version.

## Funding

Financial support was provided by the Hainan Province Science and Technology Special Fund of China (GHYF2022012).

## Conflict of interest

The authors declare that the research was conducted in the absence of any commercial or financial relationships that could be construed as a potential conflict of interest.

## Publisher’s note

All claims expressed in this article are solely those of the authors and do not necessarily represent those of their affiliated organizations, or those of the publisher, the editors and the reviewers. Any product that may be evaluated in this article, or claim that may be made by its manufacturer, is not guaranteed or endorsed by the publisher.
